# Analysis of comorbidity patterns of pregnancy and delivery complications in the female population

**DOI:** 10.3389/fgwh.2026.1824757

**Published:** 2026-06-18

**Authors:** Xiaoli Zhang, Xueer Ma, Shanshan Shan, Xiao Yao, Ziyun Gu, Yaoxiang Duan

**Affiliations:** Nursing Department, Shanghai Key Laboratory of Maternal Fetal Medicine, Shanghai Institute of Maternal Fetal Medicine and Gynecologic Oncology, Shanghai First Maternity and Infant Hospital, School of Medicine, Tongji University, Shanghai, China

**Keywords:** association rule mining, delivery complications, gestational diabetes mellitus, gestational hypertension, network analysis, pregnancy complications

## Abstract

**Background:**

Understanding the core diseases and comorbidity patterns of maternal complications during pregnancy and delivery is crucial for developing preventive strategies and improving pregnancy outcomes.

**Methods:**

This cross-sectional study investigated maternal complications during pregnancy and delivery in women who gave birth at the Shanghai First Maternity and Infant Hospital between January 2023 and November 2025. A comorbidity network of maternal complications was constructed, and core comorbidities within the network were identified on the basis of topological network indicators. The Apriori association rule algorithm was applied to identify common binary comorbidity patterns in maternal health.

**Results:**

The average age of the 15,773 female participants was 31.12 (3.53) years. The most common pregnancy-related complication was anemia during pregnancy (27.95%), whereas the most common delivery-related complication was postpartum hemorrhage (21.24%). Network analysis revealed that gestational diabetes mellitus was positioned at the center of the comorbidity network and exhibited the highest strength centrality (strength = 1.61). When separate comorbidity networks were constructed for the advanced maternal age and nonadvanced maternal age groups, no significant differences were found between the networks (*P* > 0.05). The Apriori algorithm generated 40 association rules, with the strongest association based on the lift values between “gestational diabetes mellitus and gestational hypertension” (lift = 3.868).

**Conclusion:**

The core complication in both the advanced maternal age and nonadvanced maternal age groups during pregnancy and delivery was gestational diabetes mellitus, with the core comorbidity pair being “gestational diabetes mellitus and gestational hypertension”. These findings suggest that prioritizing joint screening and integrated management of gestational diabetes mellitus and gestational hypertension may help reduce downstream complications and improve maternal outcomes.

## 1 Introduction

Pregnancy and delivery affect women and families worldwide, with at least 40 million women potentially experiencing long-term health issues related to pregnancy and delivery each year (1). Pregnancy-related and delivery-related complications play critical roles in maternal health management (2). Pregnancy complications include conditions such as gestational hypertension, gestational diabetes mellitus, and anemia during pregnancy, whereas delivery complications include postpartum hemorrhage and amniotic fluid embolism (3, 4). These complications not only increase maternal suffering and healthcare costs but also may lead to severe long-term health issues (5, 6). If gestational hypertension is not effectively managed in a timely manner, it may progress to severe preeclampsia, threatening the lives of both mothers and their infants (7, 8). The occurrence of gestational diabetes mellitus may increase the risk of maternal hemorrhage and preterm birth (9–11). Furthermore, postpartum hemorrhage increases the risk of renal failure and maternal mortality (12, 13). Therefore, understanding and preventing pregnancy and delivery complications are crucial for improving maternal and neonatal health outcomes.

Comorbidity patterns refer to the simultaneous presence of two or more diseases or health conditions in the same individual, where these diseases may interact with each other (14). In recent years, with the increasing proportion of pregnant women of advanced maternal age (AMA), both gestational and delivery complications have been gradually increasing (15, 16). These complications tend to occur simultaneously, resulting in comorbidity patterns that increase the difficulty and cost of medical interventions and pose serious threats to maternal and neonatal health (17, 18). However, recent research has largely focused on exploring the pathogenesis, influencing factors, and interventions for individual diseases, with limited research on the comorbidity patterns of pregnancy and delivery complications or the identification of core comorbidities.

Network analysis is a method used to study the relationships between elements in complex systems, enabling the visualization of associations between diseases and identifying core diseases and structural features of a disease network (19). The Apriori algorithm is a classic association rule mining technique that helps to identify comorbidity patterns by discovering frequent disease combinations and strong association rules (20). This study employed both of these methods to analyze maternal records from the Shanghai First Maternity and Infant Hospital, aiming to identify core comorbidities and the comorbidity patterns of gestational and delivery complications. The goal was to gain a deeper understanding of the comorbidity characteristics of maternal complications, providing a basis for clinical interventions and health management to improve maternal health outcomes.

## 2 Material and methods

### 2.1 Data source

In this study, electronic medical record data were collected for 15,773 women who received care at the Shanghai First Maternity and Infant Hospital, a tertiary specialized obstetrics and gynecology hospital in Shanghai, China, between January 2023 and November 2025. The cohort included women who underwent delivery (live or stillbirth) as well as those treated for pregnancy complications without delivery, such as ectopic pregnancy or early spontaneous abortion. Key information extracted from the records included age, gravidity, parity, pregnancy complication status, delivery complication status, total labor duration, and blood loss. Ethical approval for this study was obtained from the Medical Ethics Committee of the Shanghai First Maternity and Infant Hospital, School of Medicine, Tongji University (Approval No. 2024231).

### 2.2 Definitions of pregnancy and delivery complications

In this study, pregnancy and delivery complications were defined according to the standards outlined in the 10th edition of the *Obstetrics and Gynecology* textbook published by the People's Medical Publishing House of China (21). Detailed operational definitions of the major included complications, including diagnostic criteria, ascertainment window, data source, and coding rules, are provided in Supplementary Table S1. The included pregnancy complications comprised spontaneous abortion, ectopic pregnancy, excessive vomiting during pregnancy, gestational hypertension, intrahepatic cholestasis of pregnancy, acute fatty liver of pregnancy, preterm labor, and prolonged pregnancy. Additionally, pregnancy-related medical and surgical conditions included cardiac diseases, diabetes, viral hepatitis, TORCH syndrome, hematologic disorders (such as anemia and thrombocytopenia), thyroid disorders (such as hyperthyroidism and hypothyroidism), acute appendicitis, and acute pancreatitis. Delivery complications included postpartum hemorrhage, amniotic fluid embolism, and uterine rupture.

### 2.3 Statistical analyses

Data analysis and visualization were performed using R version 4.3.2. Descriptive statistics, including frequencies, percentages, and means ± standard deviations, are used to summarize the demographic characteristics and comorbidities of the women.

#### 2.3.1 Network estimation

For visualization and network analysis, the “qgraph” and “igraph” packages in R were used to generate a visual representation of the network. An undirected weighted comorbidity network was estimated using a Gaussian graphical model (GGM) with EBICglasso regularization via the “bootnet” package (22, 23). The network visualization was processed using the Fruchterman-Reingold force-directed layout algorithm, which positions nodes with higher centrality closer to the center of the network while clustering nodes with similar characteristics more tightly (24). In this network, “nodes” represent specific diseases, and “edges” indicate the co-occurrence relationships between diseases.

#### 2.3.2 Network stability

To assess the accuracy of the constructed network, the bootstrap method from the “bootnet” package was employed in this study, and the correlation stability coefficient (CS) was calculated to evaluate the stability of the network. Generally, a CS value should not fall below 0.25, and values above 0.5 are preferable (25). Additionally, bootstrap difference tests were performed to rigorously evaluate differences in network characteristics.

#### 2.3.3 Network centrality

This study used network topology metrics to assess the importance of nodes, with the following specific indicators:

- *Strength centrality:* This indicator measures the extent of a disease's involvement in the comorbidity network. Higher strength centrality indicates that a disease is more likely to be a core condition.
- *Betweenness centrality:* This indicator refers to the number of times a disease acts as a bridge on the shortest path between two other disease nodes. Higher betweenness centrality suggests a greater effect of a disease on the network.
- *Closeness centrality:* This indicator represents the reciprocal of the sum of the shortest distances from a disease to all other diseases. Higher closeness centrality indicates greater proximity to other nodes, reflecting a stronger indirect effect on other diseases.

Among these metrics, strength centrality is the most critical. When a core disease emerges or worsens, it is more likely to influence the development of other diseases, potentially activating the entire network and triggering other interconnected conditions.

#### 2.3.4 Apriori algorithm

To identify closely associated comorbidities in this study, association rule mining was applied to identify common binary comorbidity patterns in the study population (26–28). The following metrics were used:

- *Support:* This metric represents the probability of the co-occurrence of Disease A and Disease B, calculated as the number of individuals with the comorbidity pair divided by the total sample size. The formula is as follows: Support
  $$\;(A\to B)=\frac{\mathrm{Number}\;\mathrm{of}\;\mathrm{transactions}\;\mathrm{containing}\;\mathrm{both}\;A\;\;\mathrm{and}\;B\;}{\mathrm{Total}\;\;\mathrm{number}\;\mathrm{of}\;\mathrm{transactions}\;\;\mathrm{in}\;\mathrm{dataset}}$$
- *Confidence:* This metric indicates the probability that individuals with Disease A also have Disease B. The formula is as follows: Confidence
  $$(A\to B)=\frac{\mathrm{Support}\;(A\cup B)\;}{\mathrm{Support}\;(A)}$$
- *Lift:* This metric represents the ratio of the observed co-occurrence rates of Disease A and Disease B to the expected co-occurrence rate if these diseases are independent. Higher lift values suggest stronger associations. The formula is as follows: Lift
  $$(A\to B)=\frac{\mathrm{Support}\;(A\cup B)\;}{\mathrm{Support}\;(A)\;*\;\mathrm{Support}\;(B)}$$

To identify meaningful association rules, thresholds were set as follows: disease prevalence >1%, support >1%, confidence >1%, and lift >1.

#### 2.3.5 Network comparison by maternal Age

Maternal age is a critical factor influencing comorbidities in the maternal population. Therefore, the NetworkComparisonTest (NCT) was employed in this study to compare network characteristics between advanced maternal age (AMA) and non-AMA groups. NCT is a permutation-based hypothesis test that evaluates differences between two networks under the null hypothesis of structural invariance. The comorbidity networks of the AMA and non-AMA groups were compared to assess differences in network structure and overall strength. Statistical significance was set at *P* < 0.05.

## 3 Results

### 3.1 Demographic characteristics

A total of 15,773 individuals from the maternal population were included in this study, with a mean age of 31.12 years (SD = 3.53). Among these women, 4,199 (26.62%) experienced two or more pregnancy- and delivery-related complications. Primiparous women accounted for 11,519 (73.03%) of the total sample, 14,804 (93.86%) had spontaneous vaginal deliveries, and 13,873 (87.95%) utilized pain relief measures during delivery. The details are presented in Table 1.

**Table 1:** Demographic characteristics of the maternal population.

**Characteristics**	***n* (%)**	**Characteristics**	***n* (%)/Mean (SD)**
**Advanced maternal age**	**Perineal tear**		
Yes (≥35 years)	2,552 (16.18%)	None	3,816 (24.19%)
No (<35 years)	13,221 (83.82%)	Grade Ⅰ	2,152 (13.64%)
**Primiparous**		Grade Ⅱ	9,803 (62.15%)
Yes	11,519 (73.03%)	Grade Ⅲ	1 (0.01%)
No	4,254 (26.97%)	Grade Ⅳ	1 (0.01%)
**Underweight before pregnancy**	**Duration of labor (hours)**		
Yes	1,765 (11.19%)	First stage	7.44 ± 3.93
No	14,008 (88.81%)	Second stage	0.54 ± 0.43
**Overweight before pregnancy**		Third stage	0.11 ± 0.07
Yes	1,562 (9.90%)	Total labor duration	8.08 ± 4.06
No	14,211 (90.10%)	**Newborn outcomes**	
**Delivery analgesia**		1-minute Apgar score	9.10 ± 0.84
Yes	13,873 (87.95%)	5-minute Apgar score	9.71 ± 0.80
No	1,900 (12.05%)	Birth length (cm)	49.78 ± 1.48
**Mode of delivery**		Birth weight (g)	3,248.49 ± 426.41
Spontaneous vaginal	14,804 (93.86%)		
Forceps-assisted	942 (5.92%)		
Breech-assisted	27 (0.17%)		

### 3.2 Comorbidity prevalence

**Figure 1:**
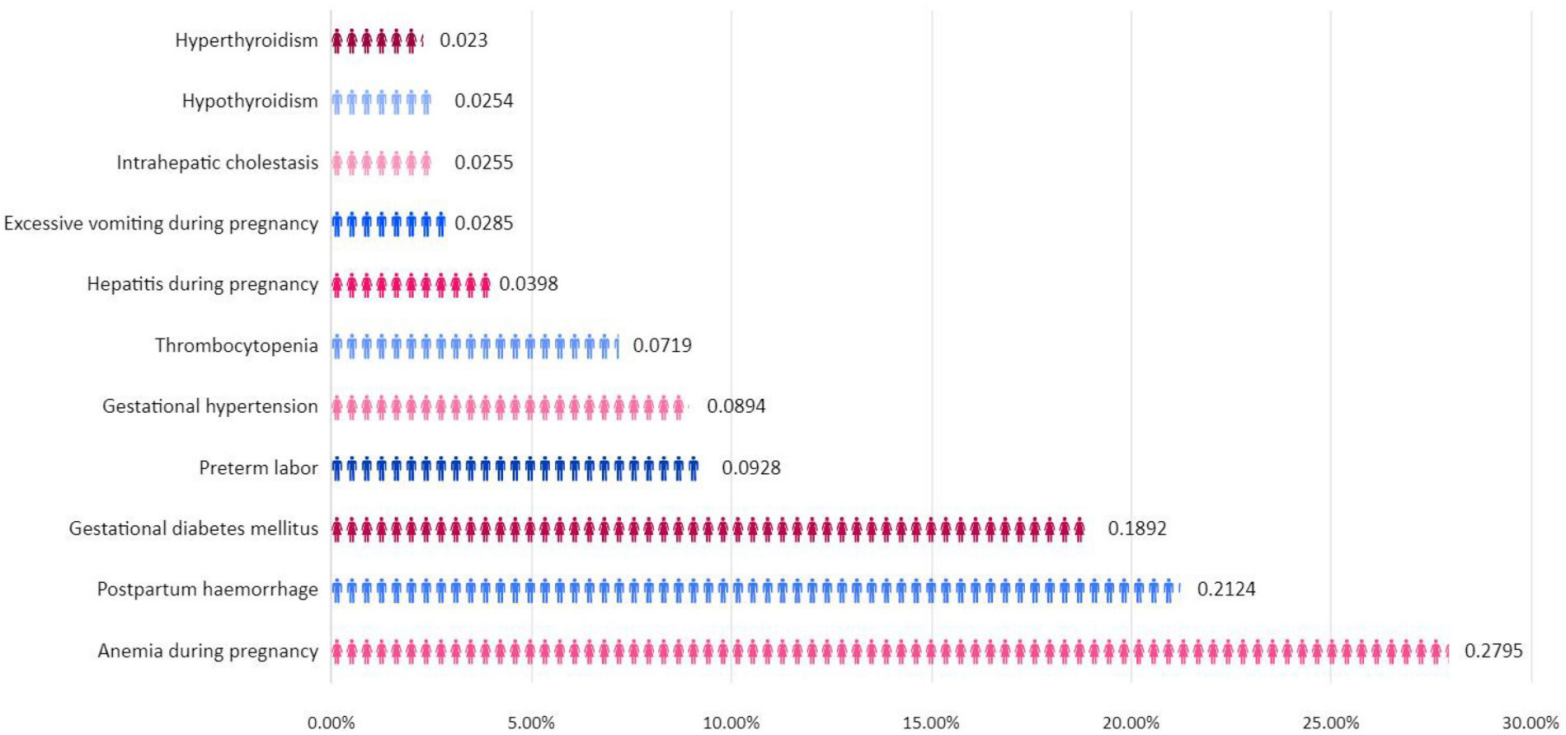
Prevalence of pregnancy- and delivery-related comorbidities in the maternal population.

The most common pregnancy-related complication was anemia during pregnancy (27.95%), followed by gestational diabetes mellitus (18.92%) and preterm labor (9.28%). The most common delivery-related complication was postpartum hemorrhage (21.24%). Conditions with an incidence rate less than 1% were excluded from the analysis, including ectopic pregnancy (0.36%), acute fatty liver of pregnancy (0.34%), pregnancy-related cardiac disease (0.22%), prolonged pregnancy (0.11%), acute appendicitis during pregnancy (0.08%), spontaneous abortion (0.06%), acute pancreatitis during pregnancy (0.05%), TORCH syndrome (<0.01%), amniotic fluid embolism (<0.01%), and uterine rupture (<0.01%). The details are presented in Figure 1.

### 3.3 Comorbidity network of the entire maternal population

**Figure 2:**
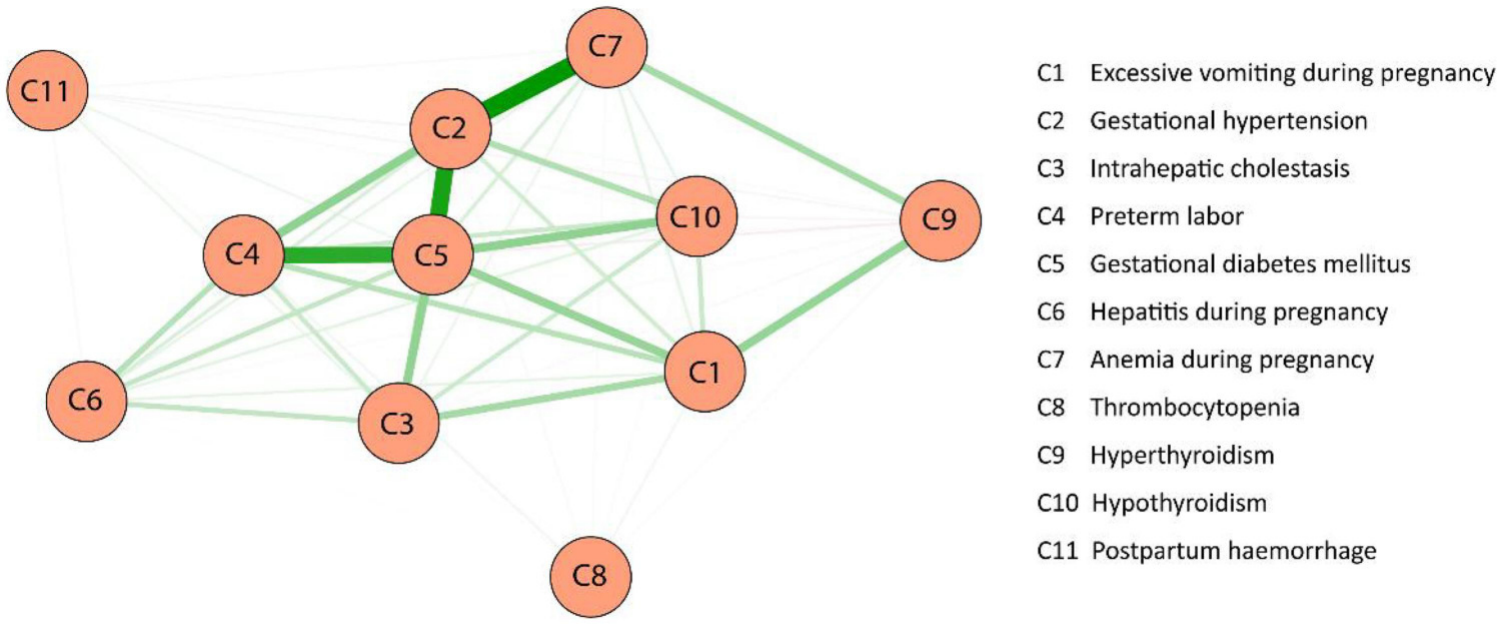
Comorbidity network of the entire maternal population.

The comorbidity relationships among the entire maternal population were visualized using a network graph. Circles (nodes) represent specific diseases, whereas the connections (edges) between nodes indicate co-occurrence relationships. The thickness of an edge reflects the frequency of co-occurrence between two diseases; thicker lines indicate a higher frequency, whereas thinner lines indicate a lower frequency. Gestational diabetes mellitus was positioned at the center of the comorbidity network, exhibiting the highest strength centrality (strength = 1.61), followed by gestational hypertension (strength = 1.59). These findings suggest that gestational diabetes mellitus not only has a relatively high prevalence but also frequently cooccurs with other diseases, indirectly increasing the likelihood of comorbidities. These findings indicate that gestational diabetes mellitus may play a pivotal role as a core disease in the maternal comorbidity network (Figure 2 and Supplementary Figure S1–S5).

### 3.4 Comorbidity network of the advanced maternal age and nonadvanced maternal age groups

In the comorbidity network of the AMA group, gestational diabetes mellitus was the most common disease (strength = 2.44), followed by gestational hypertension (strength = 2.20). In the comorbidity network of the non-AMA group, gestational diabetes mellitus was also the most common disease (strength = 1.32), followed by gestational hypertension (strength = 1.23). The comorbidity networks of both the AMA and non-AMA groups were generally consistent with the network of the entire maternal population (Supplementary Figures S6, S7). The results of the network comparison test (NCT) comparing the comorbidity network strength between the AMA and non-AMA groups were provided in Supplementary Figure S8.

### 3.5 High-Frequency comorbidity patterns

**Figure 3:**
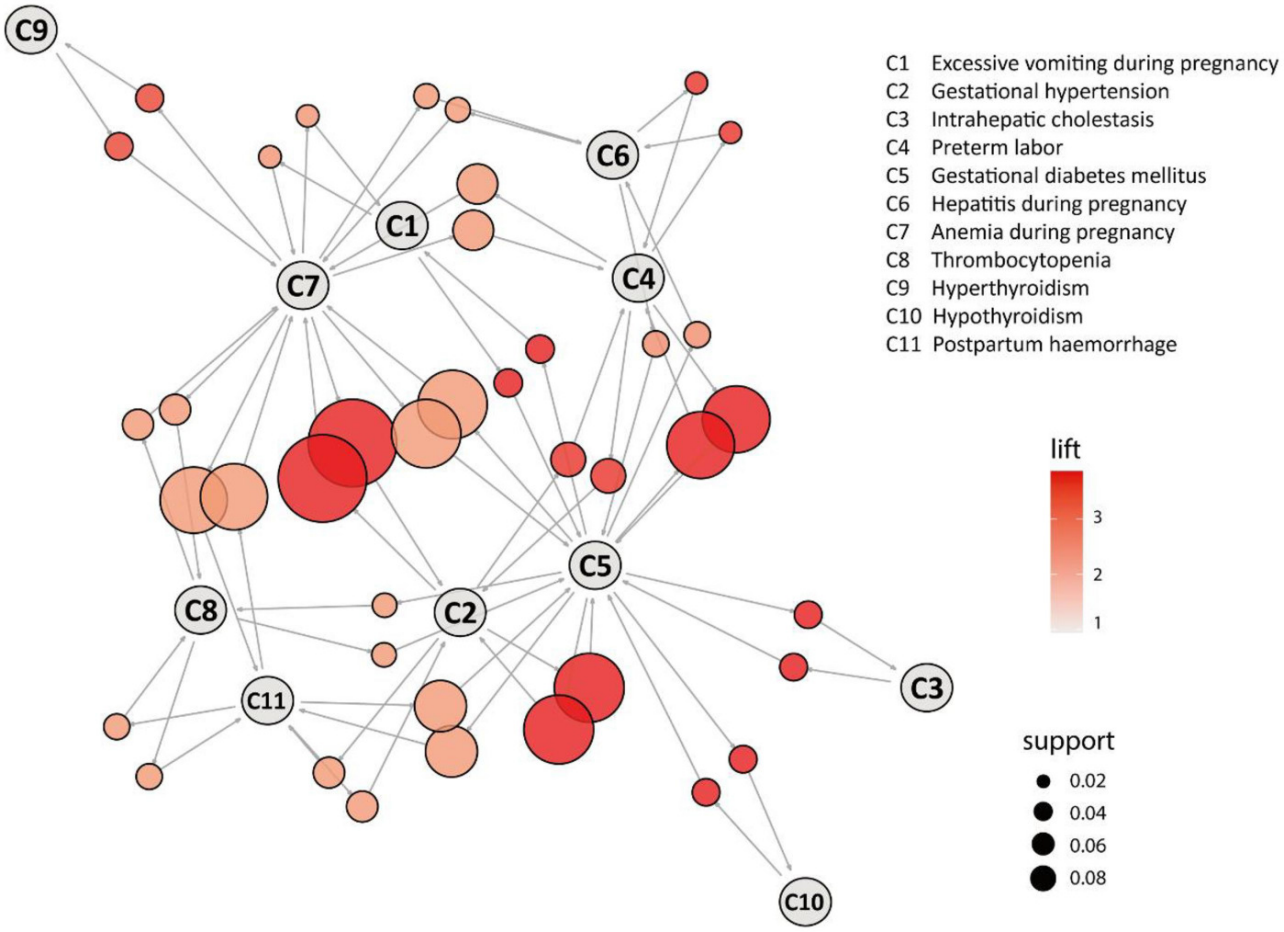
Apriori association analysis network.

In this study, the Apriori model was employed for association analysis, and the 40 identified association rules were visualized using a network graph (Figure 3). Diseases are represented as gray spheres, with the size of the spheres on the connecting lines between disease nodes reflecting the support values, where larger spheres indicate greater support. The color of the spheres represents the lift values, with darker colors indicating higher lift values. Diabetes mellitus during pregnancy and anemia during pregnancy were associated with the greatest number of comorbidity patterns, with 9 and 8 combinations, respectively.

### 3.6 High-Frequency comorbidity

**Figure 4:**
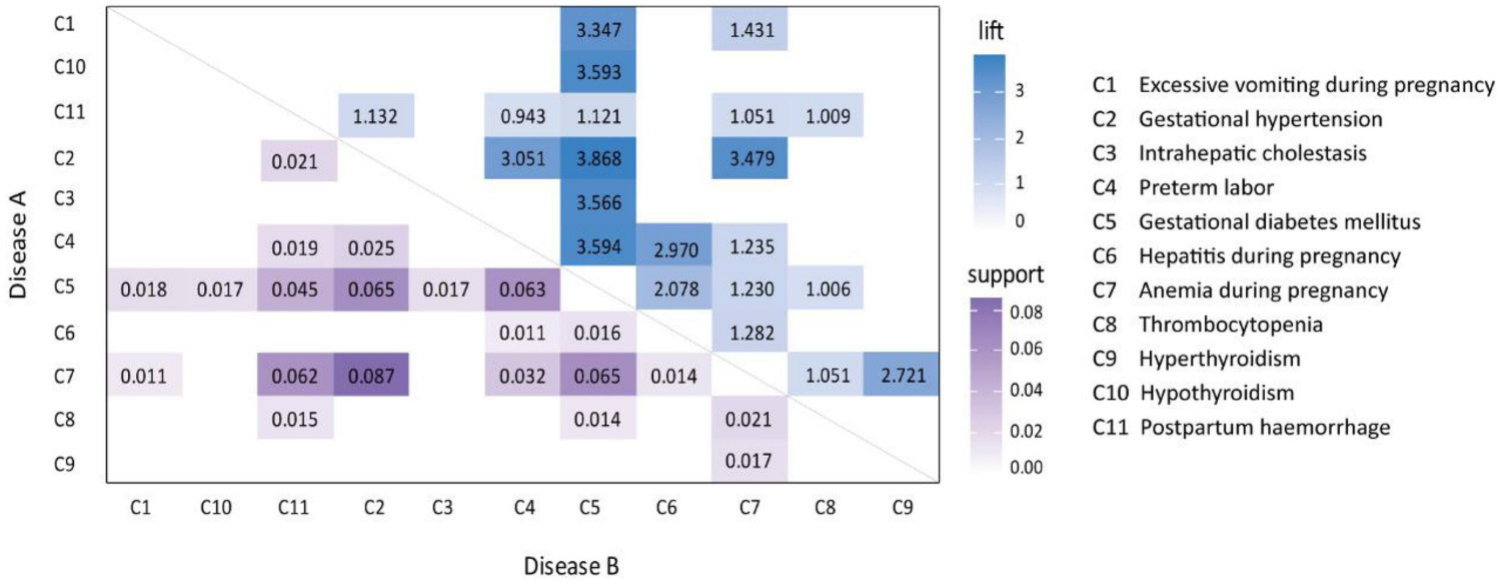
Heatmap of support and lift values for comorbidities in the maternal population.

Details on the support and lift values are provided in Figure 4. According to the lift values, the strongest association was between “gestational diabetes mellitus and gestational hypertension” (lift = 3.868), followed by “gestational diabetes mellitus and preterm labor” (lift = 3.593). When the data were sorted by support values, the most common comorbidity pair was “gestational hypertension and anemia during pregnancy” (support = 0.087).

**Figure 5:**
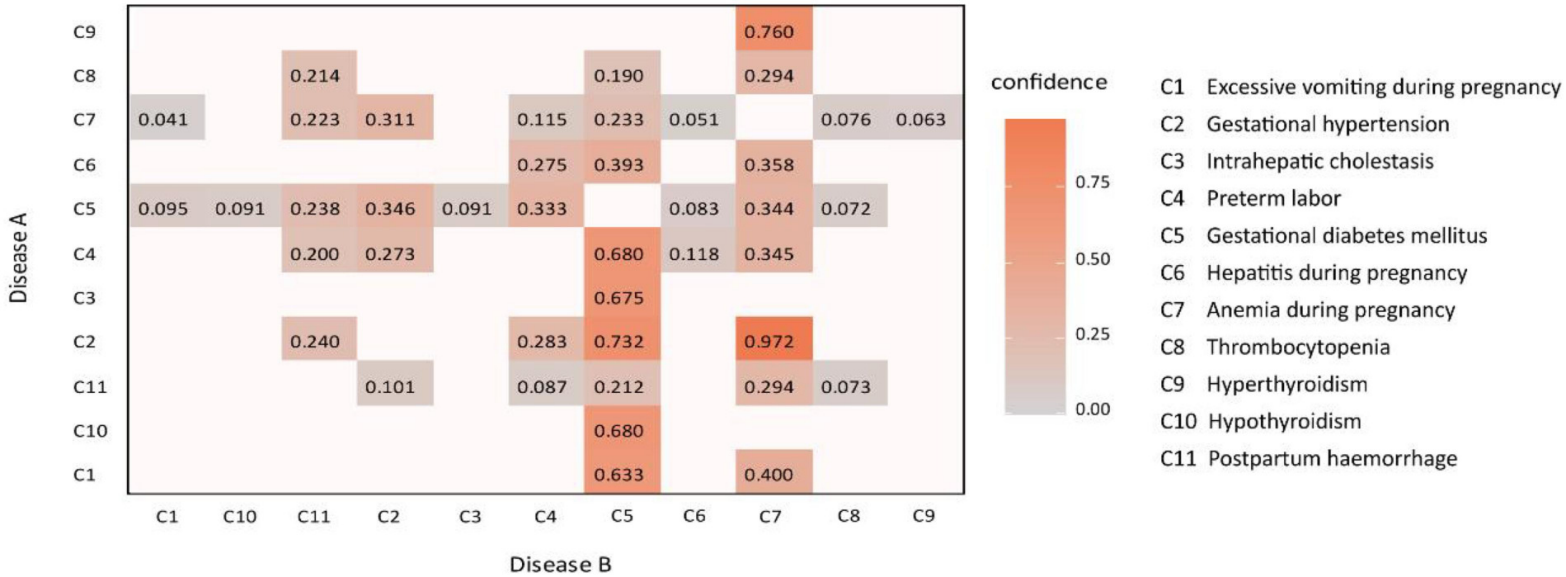
Heatmap of confidence for comorbidities in the maternal population.

Figure 5 presents the heatmap for confidence values, where higher confidence values indicate a greater probability of Disease B occurring among individuals with Disease A. Gestational diabetes mellitus, anemia during pregnancy, and gestational hypertension were commonly associated with a higher prevalence of various other pregnancy-related complications.

## 4 Discussion

The results of this study indicate that the rate of comorbid maternal conditions during pregnancy and delivery is 26.62%, which is lower than the findings of McCauley et al. (17). This difference may be attributed to the increasing health awareness among maternal populations and their heightened attention to pregnancy- and delivery-related complications. Recent studies have suggested that maternal populations of AMA tend to experience more complications than non-AMA populations do and are at a greater risk of adverse pregnancy outcomes (29–31). However, no statistically significant differences were found between the comorbidity networks of the AMA and non-AMA groups in this study. This may be because the network analysis employed in this study focused primarily on the relationships between diseases rather than on disease incidence rates. Although differences in disease prevalence existed between AMA and non-AMA groups, the overall structure of the network and the core diseases remained consistent. Currently, there is limited research on the disease patterns associated with maternal complications, and future studies are needed to validate these findings.

The results of this study also indicate that although anemia during pregnancy has the highest incidence, gestational diabetes mellitus is the core comorbidity among pregnancy and delivery-related complications. This could be due to the increased blood volume during pregnancy, which dilutes the blood, causing a relative decrease in the red blood cell count and hemoglobin levels, leading to the diagnosis of anemia during pregnancy (32, 33). However, this type of anemia is not due to insufficient hematopoietic materials or abnormal bone marrow function but rather a temporary physiological phenomenon and therefore has a minimal effect on other comorbidities (34). Gestational diabetes mellitus may share common metabolic, inflammatory, and vascular pathways with other pregnancy complications, including gestational hypertension. The microvascular changes caused by gestational diabetes mellitus lead to thickening of the small vessel endothelium and narrowing of the lumen, resulting in tissue ischemia and further increasing the risk of gestational hypertension and preeclampsia (35, 36). A systematic review (37) revealed that gestational diabetes mellitus increases the risk of adverse pregnancy outcomes, such as gestational hypertension and preterm birth. Goodman JR (38) noted that gestational diabetes mellitus increases the risk of complications for both mothers and infants, including miscarriage and delivery-related trauma. Furthermore, mothers with gestational diabetes mellitus, due to increased risks of macrosomia, obstructed labor, and birth canal injuries, are more likely to experience prolonged labor and develop postpartum hemorrhage (39–41). For future clinical practice, this highlights the importance of enhancing screening and management for gestational diabetes mellitus among pregnant women, with early interventions to reduce the risk of comorbidities. However, the present study cannot determine whether gestational diabetes mellitus directly contributes to the development of gestational hypertension.

The results of this study indicate that gestational diabetes mellitus and gestational hypertension are the core comorbidities associated with maternal pregnancy- and delivery-related complications. This finding is consistent with those of several other studies. A case‒control study revealed a strong correlation between gestational diabetes mellitus and gestational hypertension (42). Aburezq M's (43) cross-sectional study also revealed gestational hypertension as an independent risk factor for gestational diabetes mellitus. This could be due to shared pathophysiological mechanisms between the two diseases. For example, insulin resistance leads to hyperinsulinemia, which in turn causes weight gain, water and sodium retention, and increased sympathetic activity. These factors not only contribute to elevated blood glucose levels but also increase blood pressure (44, 45). Moreover, pregnant women with gestational diabetes mellitus experience increased insulin resistance, which requires higher insulin levels to maintain normal blood glucose levels (46). Hyperinsulinemia further exacerbates insulin resistance, creating a vicious cycle that increases the risk of gestational hypertension (47). Additionally, prolonged hyperglycemia in pregnant women with diabetes mellitus triggers various pathological processes, including nonenzymatic glycation, lipid metabolism changes, local hypoxia, increased reactive oxygen species synthesis, and an imbalance in inflammatory cytokine production. These processes can lead to vascular remodeling and endothelial damage, increasing maternal susceptibility to gestational hypertension (48, 49). When gestational diabetes mellitus and gestational hypertension coexist, the risk of adverse neonatal outcomes is significantly greater (50). Therefore, clinically, it is crucial to remain highly vigilant for the development of gestational hypertension when diagnosing and treating gestational diabetes mellitus so that timely and comprehensive therapeutic interventions can be implemented to improve maternal outcomes. Future research could further explore the molecular mechanisms underlying the association between these two diseases, providing a theoretical basis for the development of new combined therapeutic approaches.

## 5 Conclusion and recommendations

This study revealed the comorbidity patterns of common maternal pregnancy and delivery complications, providing new directions for the early identification and intervention of maternal complications. The findings indicate that the core comorbidity for maternal health is gestational diabetes mellitus, with the strongest comorbidity pair being gestational diabetes mellitus and gestational hypertension. In clinical practice, healthcare professionals should strengthen the comprehensive management of maternal comorbidities, particularly focusing on gestational diabetes mellitus and gestational hypertension. Future efforts can involve developing personalized screening, diagnostic, and treatment plans to increase the quality of healthcare services and improve maternal health outcomes. In terms of research, further increasing the sample size, utilizing multicenter and prospective study designs, and exploring the mechanisms, influencing factors, and interactions between different comorbidity pairs in depth are recommended, thereby providing more solid scientific evidence for optimizing maternal health management strategies.

## 6 Limitations

Several limitations should be acknowledged. First, this was a single-center, hospital-based study conducted in a tertiary obstetrics and gynecology hospital, and the study population may not fully represent the general pregnant population. Women referred to or managed in tertiary care settings may have a higher burden of complications, which may introduce selection bias. Second, all complications were identified from electronic medical records; therefore, measurement bias and coding bias may exist, particularly for conditions with variable diagnostic thresholds or incomplete clinical documentation. Third, differences in clinical management before or during hospitalization may have influenced the observed comorbidity patterns. Fourth, because of the cross-sectional nature of this study, the identified network edges and association rules should be interpreted as co-occurrence associations rather than causal pathways. The present analysis cannot determine whether one complication preceded or contributed to another, nor can it establish exposure-outcome sequences. Finally, sensitivity analyses using different Apriori thresholds or network edge-selection criteria were not performed in the current study. Future studies should further evaluate the robustness of the identified comorbidity patterns through additional sensitivity analyses, multicenter validation, and prospective study designs.

## Data Availability

The raw data supporting the conclusions of this article will be made available by the authors, without undue reservation.

## References

[1] Vogel JP Jung J Lavin T Simpson G Kluwgant D Abalos E. Neglected medium-term and long-term consequences of labour and childbirth: a systematic analysis of the burden, recommended practices, and a way forward. Lancet Glob Health. (2024) 12(2):e317–30. 10.1016/s2214-109x(23)00454-0 38070535 PMC10805007

[2] Graham W Woodd S Byass P Filippi V Gon G Virgo S. Diversity and divergence: the dynamic burden of poor maternal health. Lancet. (2016) 388(10056):2164–75. 10.1016/s0140-6736(16)31533-1 27642022

[3] Singh M Fayaz FFA Wang J Wambua S Subramanian A Reynolds JA. Pregnancy complications and autoimmune diseases in women: systematic review and meta-analysis. BMC Med. (2024) 22(1):339. 10.1186/s12916-024-03550-5 39183290 PMC11346028

[4] Guglielminotti J Landau R Wong CA Li G. Criticality of maternal complications during childbirths. J Patient Saf. (2020) 16(4):e273–7. 10.1097/PTS.0000000000000511 29985887

[5] Law A McCoy M Lynen R Curkendall SM Gatwood J Juneau PL. The prevalence of complications and healthcare costs during pregnancy. J Med Econ. (2015) 18(7):533–41. 10.3111/13696998.2015.1016229 25714263

[6] McNestry C Killeen SL Crowley RK McAuliffe FM. Pregnancy complications and later life women’s health. Acta Obstet Gynecol Scand. (2023) 102(5):523–31. 10.1111/aogs.14523 36799269 PMC10072255

[7] Sweeney LC Lundsberg LS Culhane JF Partridge C Son M. Co-existing chronic hypertension and hypertensive disorders of pregnancy and associated adverse pregnancy outcomes. J Matern Fetal Neonatal Med. (2024) 37(1):2305675. 10.1080/14767058.2024.2305675 38290827

[8] Liu C Liu X. Hypertensive disorders of pregnancy: causes, diagnosis, complications, and management strategies. MEDS Clinical Medicine. (2024) 5(1):61–70. 10.23977/medsc.2024.050111

[9] Sweeting A Hannah W Backman H Catalano P Feghali M Herman WH. Epidemiology and management of gestational diabetes. Lancet. (2024) 404(10448):175–92. 10.1016/s0140-6736(24)00825-0 38909620

[10] Ugwudike B Kwok M. Update on gestational diabetes and adverse pregnancy outcomes. Curr Opin Obstet Gynecol. (2023) 35(5):453–9. 10.1097/gco.0000000000000901 37560815

[11] McIntyre HD Catalano P Zhang C Desoye G Mathiesen ER Damm P. Gestational diabetes mellitus. Nat Rev Dis Primers. (2019) 5(1):47. 10.1038/s41572-019-0098-8 31296866

[12] Almutairi WM. Literature review: physiological management for preventing postpartum hemorrhage. Healthcare (Basel). (2021) 9(6):658. 10.3390/healthcare9060658 34073073 PMC8227540

[13] Hofer S Blaha J Collins PW Ducloy-Bouthors A-S Guasch E Labate F. Haemostatic support in postpartum haemorrhage: a review of the literature and expert opinion. Eur J Anaesthesiol. (2023) 40(1):29–38. 10.1097/eja.0000000000001744 36131564 PMC9794135

[14] Valderas JM Starfield B Sibbald B Salisbury C Roland M. Defining comorbidity: implications for understanding health and health services. Ann Fam Med. (2009) 7(4):357–63. 10.1370/afm.983 19597174 PMC2713155

[15] Correa-de-Araujo R Yoon SSS. Clinical outcomes in high-risk pregnancies due to advanced maternal age. J Womens Health (Larchmt). (2021) 30(2):160–7. 10.1089/jwh.2020.8860 33185505 PMC8020515

[16] Saccone G Gragnano E Ilardi B Marrone V Strina I Venturella R. Maternal and perinatal complications according to maternal age: a systematic review and meta-analysis. Int J Gynaecol Obstet. (2022) 159(1):43–55. 10.1002/ijgo.14100 35044694 PMC9543904

[17] McCauley M Zafar S Van Den Broek N. Maternal multimorbidity during pregnancy and after childbirth in women in low- and middle-income countries: a systematic literature review. BMC Pregnancy Childbirth. (2020) 20(1):637. 10.1186/s12884-020-03303-1 33081734 PMC7574312

[18] Metcalfe A Wick J Ronksley P. Racial disparities in comorbidity and severe maternal morbidity/mortality in the United States: an analysis of temporal trends. Acta Obstet Gynecol Scand. (2018) 97(1):89–96. 10.1111/aogs.13245 29030982

[19] Vulliard L Menche J. Complex networks in health and disease. In: Barabási AL, editor. Systems Medicine. London: Academic Press (2020). 10.1016/B978-0-12-801238-3.11640-X

[20] Ma H Ding J Liu M Liu Y. Connections between Various disorders: combination pattern mining using apriori algorithm based on diagnosis information from electronic medical records. Biomed Res Int. (2022) 2022:2199317. 10.1155/2022/2199317 35601156 PMC9122731

[21] Obstetrics and Gynecology. Beijing, China: People’s Medical Publishing House of China. Beijing: People's Medical Publishing House (2024).

[22] Hevey D. Network analysis: a brief overview and tutorial. Health Psychol Behav Med. (2018) 6(1):301–28. 10.1080/21642850.2018.1521283 34040834 PMC8114409

[23] Shutta KH De Vito R Scholtens DM Balasubramanian R. Gaussian Graphical models with applications to omics analyses. Stat Med. (2022) 41(25):5150–87. 10.1002/sim.9546 36161666 PMC9672860

[24] Gajdoš P Ježowicz T Uher V Dohnálek P. A parallel fruchterman–reingold algorithm optimized for fast visualization of large graphs and swarms of data. Swarm Evol Comput. (2016) 26:56–63. 10.1016/j.swevo.2015.07.006

[25] Epskamp S Fried EI. A tutorial on regularized partial correlation networks. Psychol Methods. (2018) 23(4):617. 10.1037/met0000167 29595293

[26] Dehghani M Yazdanparast Z. Discovering the symptom patterns of COVID-19 from recovered and deceased patients using apriori association rule mining. Inform Med Unlocked. (2023) 42:101351. 10.1016/j.imu.2023.101351

[27] Luna JM Ondra M Fardoun HM Ventura S. Optimization of quality measures in association rule mining: an empirical study. Int J Comput Intell Syst. (2018) 12(1):59–78. 10.2991/ijcis.2018.25905182

[28] Deora CS Arora S Makani Z. Comparison of interestingness measures: support-confidence framework versus lift-irule framework. Int J Eng Res Appl. (2013) 3(2):208–15.

[29] Lean SC Derricott H Jones RL Heazell AEP. Advanced maternal age and adverse pregnancy outcomes: a systematic review and meta-analysis. PLoS One. (2017) 12(10):e0186287. 10.1371/journal.pone.0186287 29040334 PMC5645107

[30] Pinheiro RL Areia AL Mota Pinto A Donato H. Advanced maternal age: adverse outcomes of pregnancy, A meta-analysis. Acta Med Port. (2019) 32(3):219–26. 10.20344/amp.11057 30946794

[31] Zhou Y Yin S Sheng Q Yang J Liu J Li H. Association of maternal age with adverse pregnancy outcomes: a prospective multicenter cohort study in China. J Glob Health. (2023) 13:04161. 10.7189/jogh.13.04161 38038697 PMC10691438

[32] Azab AE Albasha MO Jbireal J El Hemady SY. Haematological changes during pregnancy: insight into anaemia, leukocytosis, and thrombocytopenia. East Afr Sch J Med Sci. (2020) 3(5):185–92. 10.36349/EASMS.2020.v03i05.050

[33] Karami M Chaleshgar M Salari N Akbari H Mohammadi M. Global prevalence of Anemia in pregnant women: a comprehensive systematic review and meta-analysis. Matern Child Health J. (2022) 26(7):1473–87. 10.1007/s10995-022-03450-1 35608810

[34] Stanley AY Wallace JB Hernandez AM Spell JL. Anemia in pregnancy: screening and clinical management strategies. Am J Matern Child Nurs. (2022) 47(1):25–32. 10.1097/nmc.0000000000000787 34860784

[35] Stanhewicz AE Nuckols VR Pierce GL. Maternal microvascular dysfunction during preeclamptic pregnancy. Clin Sci (Lond. (2021) 135(9):1083–101. 10.1042/cs20200894 33960392 PMC8214810

[36] Mota RI Morgan SE Bahnson EM. Diabetic vasculopathy: macro and microvascular injury. Curr Pathobiol Rep. (2020) 8(1):1–14. 10.1007/s40139-020-00205-x 32655983 PMC7351096

[37] Malaza N Masete M Adam S Dias S Nyawo T Pheiffer C. A systematic review to compare adverse pregnancy outcomes in women with pregestational diabetes and gestational diabetes. Int J Environ Res Public Health. (2022) 19(17):10846. 10.3390/ijerph191710846 36078559 PMC9517767

[38] Goodman JR. Diabetes Mellitus in pregnancy. Neoreviews. (2023) 24(3):e144–57. 10.1542/neo.24-3-e144 36854843

[39] Muche AA Olayemi OO Gete YK. Effects of gestational diabetes mellitus on risk of adverse maternal outcomes: a prospective cohort study in northwest Ethiopia. BMC Pregnancy Childbirth. (2020) 20(1):73. 10.1186/s12884-020-2759-8 32013909 PMC6998275

[40] Ye W Luo C Huang J Li C Liu Z Liu F. Gestational diabetes mellitus and adverse pregnancy outcomes: systematic review and meta-analysis. Br Med J. (2022) 377:e067946. 10.1136/bmj-2021-067946 35613728 PMC9131781

[41] Hivert M-F Backman H Benhalima K Catalano P Desoye G Immanuel J. Pathophysiology from preconception, during pregnancy, and beyond. Lancet. (2024) 404(10448):158–74. 10.1016/s0140-6736(24)00827-4 38909619

[42] Bryson CL Ioannou GN Rulyak SJ Critchlow C. Association between gestational diabetes and pregnancy-induced hypertension. Am J Epidemiol. (2003) 158(12):1148–53. 10.1093/aje/kwg273 14652299

[43] Aburezq M AlAlban F Alabdulrazzaq M Badr H. Risk factors associated with gestational diabetes mellitus: the role of pregnancy-induced hypertension and physical inactivity. Pregnancy Hypertens. (2020) 22:64–70. 10.1016/j.preghy.2020.07.010 32745722

[44] Mastrogiannis DS Spiliopoulos M Mulla W Homko CJ. Insulin resistance: the possible link between gestational diabetes mellitus and hypertensive disorders of pregnancy. Curr Diab Rep. (2009) 9(4):296–302. 10.1007/s11892-009-0046-1 19640343

[45] Plows JF Stanley JL Baker PN Reynolds CM Vickers MH. The pathophysiology of gestational diabetes Mellitus. Int J Mol Sci. (2018) 19(11):3342. 10.3390/ijms19113342 30373146 PMC6274679

[46] Sweeting A Wong J Murphy HR Ross GP. A clinical update on gestational diabetes Mellitus. Endocr Rev. (2022) 43(5):763–93. 10.1210/endrev/bnac003 35041752 PMC9512153

[47] Kampmann U Knorr S Fuglsang J Ovesen P. Determinants of maternal insulin resistance during pregnancy: an updated overview. J Diabetes Res. (2019) 2019:5320156. 10.1155/2019/5320156 31828161 PMC6885766

[48] Kornacki J Gutaj P Kalantarova A Sibiak R Jankowski M Wender-Ozegowska E. Endothelial dysfunction in pregnancy complications. Biomedicines. (2021) 9(12):1756. 10.3390/biomedicines9121756 34944571 PMC8698592

[49] Ottanelli S Napoli A Festa C Clemenza S Mecacci F. Hypertension and preeclampsia in pregnancy complicated by diabetes. In: McIntyre D, Hod M, editors. Gestational Diabetes. Vol 28. Basel: Karger Publishers (2020). p. 171–82.

[50] Lin X Zhou L Si S Cheng H Alifu X Qiu Y. Association of the comorbidity of gestational diabetes mellitus and hypertension disorders of pregnancy with birth outcomes. Front Endocrinol (Lausanne. (2024) 15:1468820. 10.3389/fendo.2024.1468820 39726848 PMC11669500

