# Supplementary material for: Analysis of comorbidity patterns of pregnancy and delivery complications in the female population
Source: Front Glob Womens Health. 2026 Jun 18;7:1824757. doi: 10.3389/fgwh.2026.1824757 (PMC13323245; doi:10.3389/fgwh.2026.1824757)
Supplement: Supplementary file 1 [file Datasheet1.docx]

**SUPPLEMENT**

**FIGURE S1** Centrality Metrics of the Comorbidity Network for the Entire Maternal Population

**FIGURE S2** Bootstrapped Confidence Intervals of Estimated Edge Weights for the Network

**FIGURE S3** Node Stability of the Comorbidity Network for the Entire Maternal Population

**FIGURE S4** Node Difference Test

**FIGURE S5** Edge Difference Test in Symptom Network Analysis

**FIGURE S6** Comorbidity Network Diagram of the Advanced (a) and Nonadvanced (b) Maternal Age Groups

**FIGURE S7** Centrality Metrics of the Comorbidity Networks for the Advanced (a) and Nonadvanced (b) Maternal Age Groups

**FIGURE S8** Comparison of Comorbidity Network Strength between the Advanced Maternal Age and Nonadvanced Maternal Age Groups


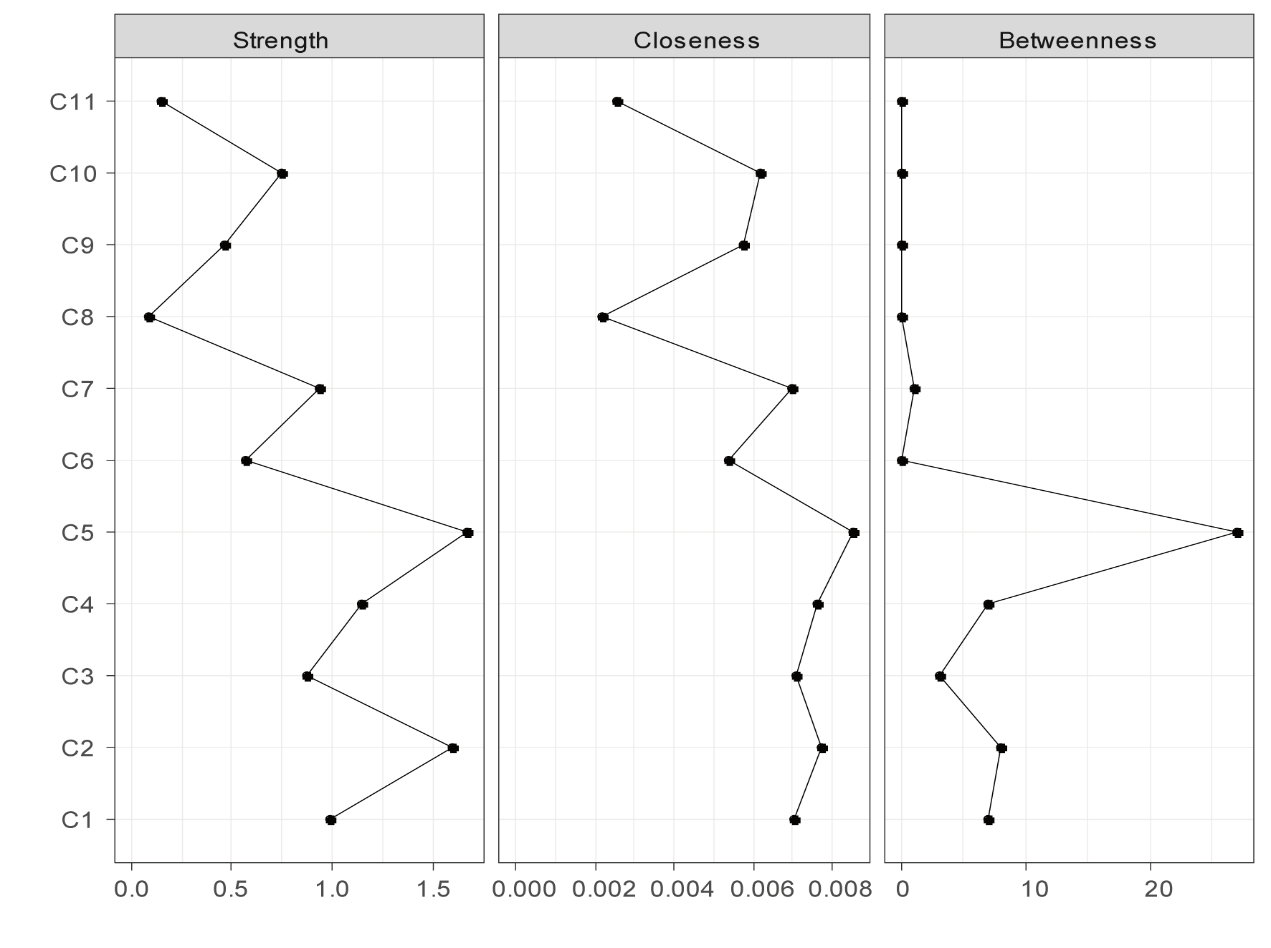


**FIGURE S1** Centrality Metrics of the Comorbidity Network for the Entire Maternal Population

**Network Accuracy and Stability Analysis**

The weighted values of the edges in this sample were in close alignment with the 95% confidence intervals of the bootstrap method, as shown in FIGURE S2, indicating that the edge weights had sufficient accuracy. The stability analysis of the symptom network revealed that the stability coefficient of the bridge strength correlation was 0.75, indicating good network stability, as detailed in FIGURE S3.


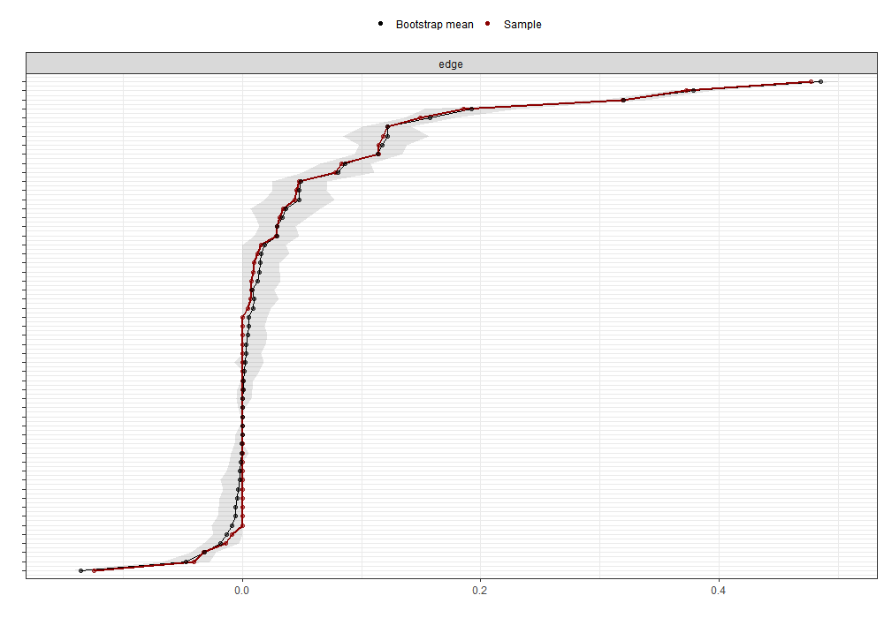


**FIGURE S2** Bootstrapped Confidence Intervals of Estimated Edge Weights for the Network


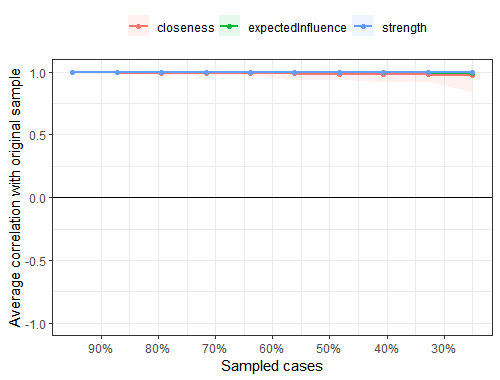


**FIGURE S3** Node Stability of the Comorbidity Network for the Entire Maternal Population

**Node and Edge Difference Test**

The bootstrap method was employed in this study to test whether the centrality of different nodes (FIGURE S4) and the edge weights of different edges (FIGURE S5) differed significantly. According to the null hypothesis, if zero lies within the confidence interval, the difference in node centrality or edge weights is not significant (represented by the gray blocks in the figure). The bootstrap difference test revealed that most of the comparisons between edge weights were statistically significant.


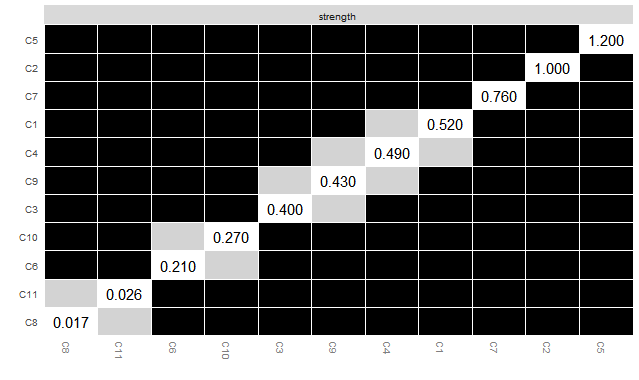


**FIGURE S4** Node Difference Test


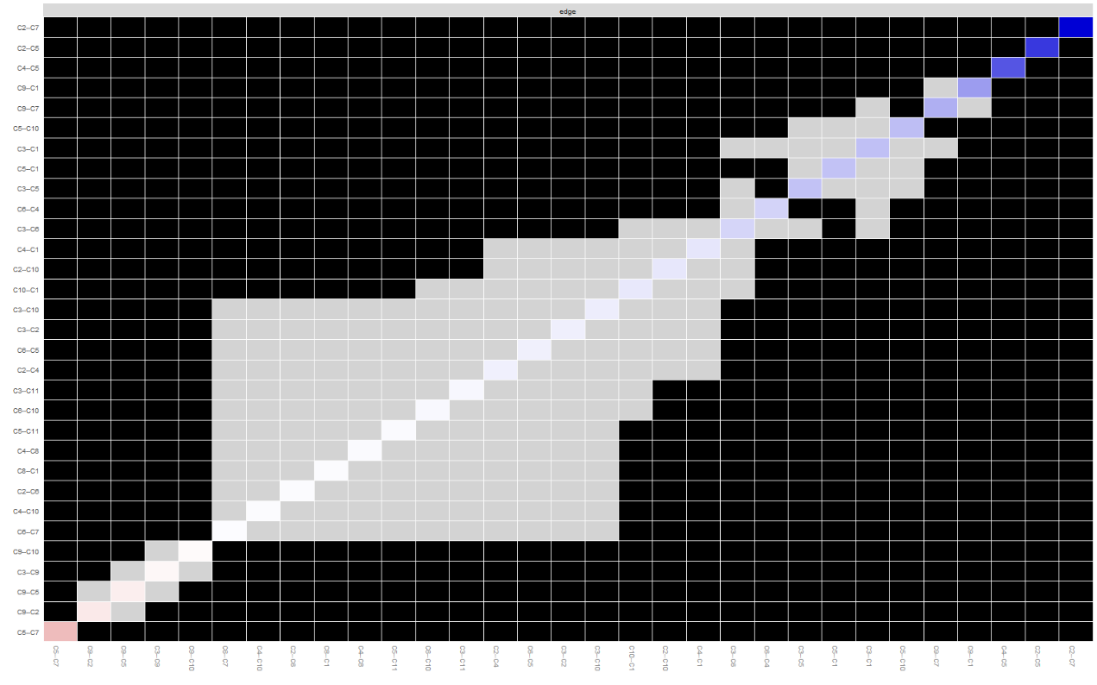


**FIGURE S5** Edge Difference Test in Symptom Network Analysis


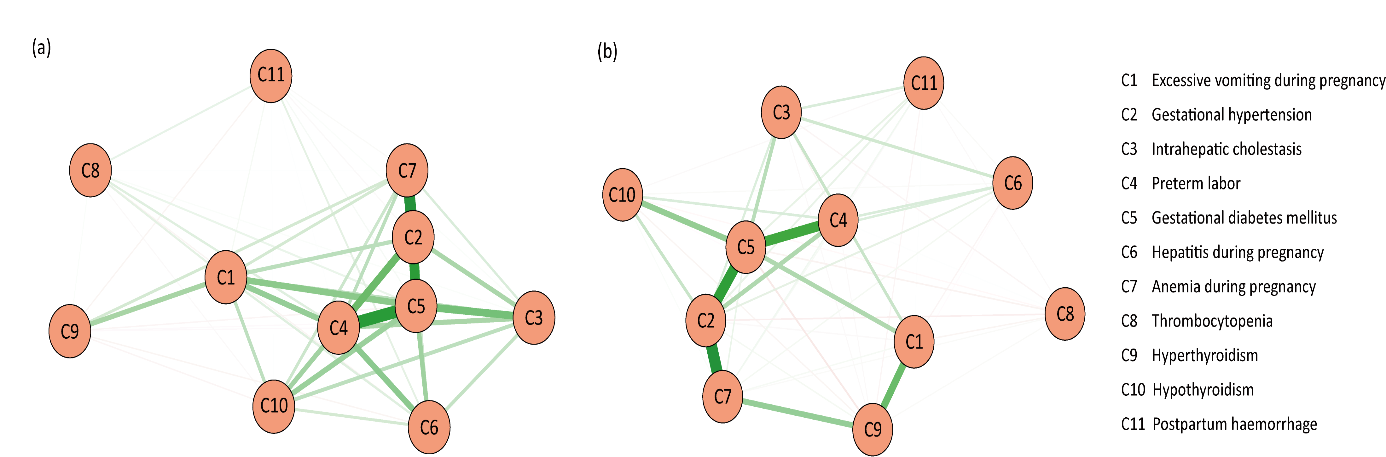


**FIGURE S6** Comorbidity Network Diagram of the Advanced (a) and Nonadvanced (b) Maternal Age Groups


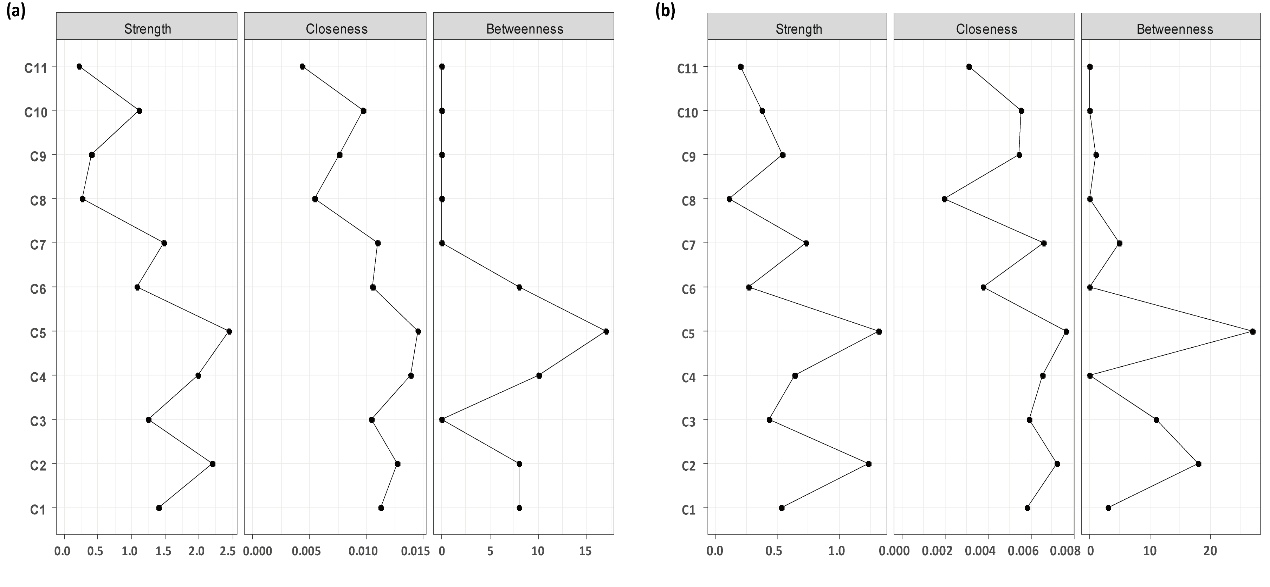


**FIGURE S7** Centrality Metrics of the Comorbidity Networks for the Advanced (a) and Nonadvanced (b) Maternal Age Groups

**Comparison of Comorbidity Network Strength between the Advanced Maternal Age and Nonadvanced Maternal Age Groups**

The results of the network comparison test (NCT) indicated no statistically significant differences in the average strength of the comorbidity networks between the AMA and non-AMA groups (*p* > 0.05), as shown in FIGURE S 8. Plot (a), which represents the bootstrap values for the maximum difference in edge weights (based on 1000 permutations), showed no significant difference (M = 1.98, *p* = 0.09). Similarly, for the bootstrap value of the difference in global network strength, plot (b) showed no significant difference (network strength in the AMA group: 39.62; network strength of in non-AMA group: 29.43; S = 10.19, *p* = 0.09). Furthermore, the invariance of edge weights was evaluated via a permutation test, which produced p values for each pairwise edge comparison. All p values were greater than 0.05, indicating that there were no significant differences between the two groups (FIGURE S8).


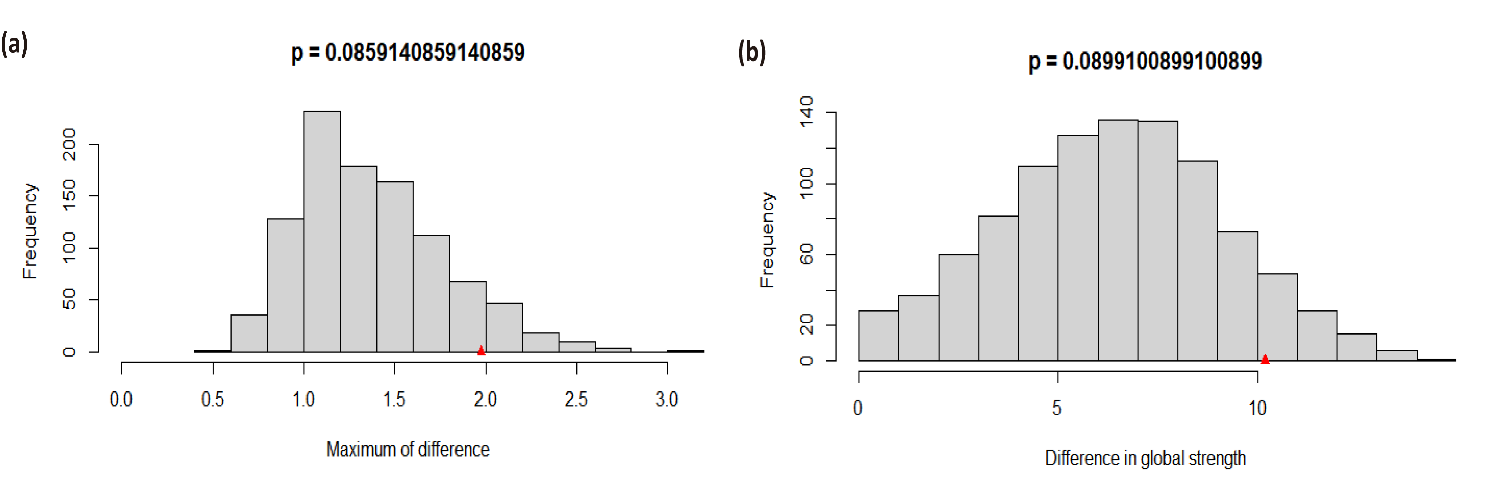


**FIGURE S8** Comparison of Comorbidity Network Strength between the Advanced Maternal Age and Nonadvanced Maternal Age Groups

(a): A plot of the bootstrap value of the maximum difference in any of the edge weights (1000 permutations).

(b): A plot of the bootstrap value of the difference in network global strength.
