# Supplementary material for: Analysis of comorbidity patterns of pregnancy and delivery complications in the female population
Source: Front Glob Womens Health. 2026 Jun 18;7:1824757. doi: 10.3389/fgwh.2026.1824757 (PMC13323245; doi:10.3389/fgwh.2026.1824757)
Supplement: Supplementary file 2 [file Datasheet2.doc]

**TABLE S1:** Operational Definitions and Diagnostic Criteria for Pregnancy- and Delivery-Related Complications

| **Complication** | **Diagnostic criteria** | **Time window** | **Data source** | **Coding rule** |
| --- | --- | --- | --- | --- |
| Gestational diabetes mellitus | Defined as either an EMR-recorded diagnosis of GDM or at least one abnormal value on a 75-g oral glucose tolerance test: fasting plasma glucose ≥5.1 mmol/L, 1-h plasma glucose ≥10.0 mmol/L, or 2-h plasma glucose ≥8.5 mmol/L. | 24–28 gestational weeks or later | EMR diagnosis/laboratory record | coded as present/absent |
| Gestational hypertension | New-onset hypertension after 20 gestational weeks, defined as systolic blood pressure ≥140 mmHg and/or diastolic blood pressure ≥90 mmHg on at least two measurements, without proteinuria or maternal organ dysfunction suggestive of preeclampsia. | ≥20 gestational weeks to delivery | EMR diagnosis/vital signs | coded as present/absent |
| Anemia during pregnancy | Defined as hemoglobin concentration <110 g/L during pregnancy, or an EMR-recorded diagnosis of anemia during pregnancy. | during pregnancy | laboratory record/diagnosis | coded as present/absent |
| Postpartum hemorrhage | Defined as estimated or quantified blood loss ≥500 mL after vaginal delivery or ≥1000 mL after cesarean delivery within 24 h after delivery, or an EMR-coded diagnosis of postpartum hemorrhage. | within 24 h after delivery, or according to hospital coding | delivery record | coded as present/absent |
| Preterm labor | Defined as delivery before 37 completed gestational weeks, regardless of the mode of delivery or indication for delivery. | At delivery | EMR | coded as present/absent |
